# Using Risk Assessment to Improve Screening for Albuminuria among US Adults without Diabetes

**DOI:** 10.1007/s11606-024-09185-9

**Published:** 2024-11-18

**Authors:** Jennifer L. Bragg-Gresham, Surekha Annadanam, Brenda Gillespie, Yiting Li, Neil R. Powe, Rajiv Saran

**Affiliations:** 1 Department of Internal Medicine, Division of Nephrology, University of Michigan, Ann Arbor, MI USA; 2 Department of Hospital Medicine, Cleveland Clinic, Cleveland, OH USA; 3 Department of Biostatistics, School of Public Health, University of Michigan, Ann Arbor, MI USA; 4 Department of Medicine, University of California, San Francisco and Priscilla Chan and Mark Zuckerberg San Francisco General Hospital, San Francisco, CA USA; 5 Department of Epidemiology, University of Michigan, School of Public Health, Ann Arbor, MI USA

## Abstract

**Background:**

Guidelines currently recommend annual screening for albuminuria only among persons with diabetes mellitus (DM). There is no guidance about albuminuria screening in those with other important risk factors for chronic kidney disease (CKD), such as hypertension and/or family history of kidney disease. We sought to create a risk score that predicts the likelihood of albuminuria in adults without diabetes to prompt earlier detection and management of CKD.

**Methods:**

Data from 44,322 participants without diabetes, aged 18 + years from the National Health and Nutrition Examination Surveys 1999–2020 were analyzed. Survey-weighted logistic regression was used to assess associations between individual characteristics and presence of albuminuria (urinary albumin to creatinine ratio [UACR] ≥ 30 mg/g), including interaction terms, in three separate models. The sample was divided equally into development and validation data sets. C-statistics were used to assess model fit.

**Results:**

The prevalence of albuminuria was 9.7% in the US adult population. Higher odds of albuminuria among the non-diabetic population were observed in females, non-Hispanic Black, and smokers, as well as those with low eGFR, hypertension, cardiovascular disease, prediabetes, low HDL cholesterol, and high uric acid levels. Age showed a J-shaped relationship with albuminuria, with lowest odds for ages 25–64 years. The C-statistic was 0.756 for the developmental and 0.752 for the validation set of the final model. Using this model, screening individuals with a predicted probability of ≥ 5% would capture 85% of individuals with albuminuria.

**Conclusions:**

These results suggest that it may be helpful to use a risk score framework for albuminuria screening in people without DM to encourage earlier detection and management of CKD. Longitudinal studies are warranted to confirm this approach along with evaluation of its cost effectiveness.

**Supplementary Information:**

The online version contains supplementary material available at 10.1007/s11606-024-09185-9.

## INTRODUCTION

Chronic kidney disease (CKD) is often asymptomatic except in its advanced stages and requires laboratory testing both for diagnosis and prognostic assessment. The absence of clinical manifestations in early stages leads to delays in diagnosis and initiation of appropriate therapies that could slow progression towards kidney failure or other complications. Earlier detection of CKD through appropriate screening and institution of evidence-based interventions has the potential to prevent further kidney damage and progressive loss of kidney function.^1–3^

Albuminuria, an early biomarker of CKD and cardiovascular disease, often precedes the loss of kidney function as assessed by declining glomerular filtration rate (GFR).^4^ One of the first studies to demonstrate this relationship was the PREVEND study (Prevention of Renal and Vascular End Stage Disease Intervention Trial; 1998 – 2003) which showed in a prospective cohort of approximately 250 subjects that elevated urinary albumin excretion at baseline was independently predictive of developing an impaired GFR.^5,6^ Since then, larger studies from around the world have supported the prognostic value of albuminuria throughout the progression of chronic kidney disease to kidney failure, cardiovascular events, and mortality. These studies have fueled interest in understanding the value of testing for albuminuria in screening for CKD.^7–11^

Screening for albuminuria has been extensively studied in diabetic populations and well-established guidelines have aided in earlier detection and treatment of CKD in individuals with diabetes.^12,13^ In those without diabetes, recommendations vary. For non-diabetic patients with hypertension, the 2017 American College of Cardiology/American Heart Association “recommends” routine urine dipstick testing, while the 2018 European Society of Cardiology/European Society of Hypertension “recommends” urine albumin to creatinine ratio (ACR) testing for all patients with hypertension as well as annual ACR testing in patients with CKD.^14,15^ For patients without hypertension or diabetes, the United States Preventive Services Task Force concluded that there was insufficient evidence to assess the benefits and risks of screening for CKD. Similarly, the American College of Physicians (ACP) published a weak recommendation given the low-quality of evidence and recommended against screening for CKD without risk factors for CKD.^16^

Given that screening the entire population would be expensive and yield modest returns, we sought to create an optimized risk model that would predict the likelihood of albuminuria among patients without diabetes. Ultimately, the goal of such a risk model would be to serve as a clinical decision tool to help clinicians determine whether to screen a given non-diabetic patient for albuminuria. Widespread implementation of this tool could facilitate more selective testing of individuals at high risk of albuminuria to improve detection of albuminuria and as a result CKD, in this population.

## METHODS

### Data Source and Study Sample

The National Health and Nutrition Examination Survey (NHANES)^26^ is a nationally representative survey that uses a complex survey design to sample adults and children in the United States on a biannual basis. We examined data from 49,796 US adults, 18 years or older, who participated in NHANES^26^ between 1999 and March 2020. The primary analysis focused on the sample of 44,322 participants who did not meet criteria for diabetes mellitus, defined as a hemoglobin A1c ≥ 6.5% and/or the participant responding positively to the question “Other than during pregnancy, have you ever been told by a doctor or health professional that you have diabetes or sugar diabetes?”.

During the 2009–2010 NHANES cycle, two measurements of albuminuria were collected on approximately half of the sample. We have analyzed this smaller cohort (*n* = 4,863) as a sensitivity analysis of our findings using only a single urine measurement.

### Predictive Variables

Albuminuria was defined as urine albumin/creatinine ratio ≥ 30 mg/g. In the cohort with two measurements per participant, both estimates of UACR ≥ 30 mg/g were required for defining albuminuria.

Demographic data include age categories (18–24 years, 25–64 years, 65–74 years, and 75 + years); ages 25 to 64 was employed as reference, as model estimates for individuals in this age range were shown to be consistent. Other variables included sex, race, and ethnicity (non-Hispanic White, non-Hispanic Black, Mexican American, Other Hispanic, and Other non-Hispanic). Household income was missing in a subset of the data (21.9%), but was otherwise categorized as < 45 K, 45-75 K, and 75 K + . Clinical variables included obesity (body mass index (BMI) ≥ 30 kg/m^2^, prediabetes (5.7 ≤ HbA1c < 6.5, low eGFR (CKD EPI eGFR < 60 ml/min.1.73m^2^), hypertension, uncontrolled high blood pressure (> 140/90 mmHg), self-reported cardiovascular disease, low HDL (< 30 mg/dl in males and < 40 mg/dl in females), high uric acid (female: uric acid > 6.1 mg/dl; male: uric acid > 7.2 mg/dl), and a history of kidney stones. Socioeconomic variables examined included: any college vs. no college education, smoking status, health insurance, moderate physical activity, vigorous physical activity. Medications examined were angiotensin-converting enzymes (ACEs) inhibitor, angiotensin II receptor blockers (ARBs), and statin. Clinical variables investigated that were continuous measures included BMI (per 1 kg/m^2^), total cholesterol (per 50 mg/dl), and high-density lipoprotein (per 10 mg/dl).

### Statistical Analysis

Baseline characteristics of participants were evaluated using descriptive statistics and employing univariate logistic regression to assess the association of individual characteristics with albuminuria using odds ratios (OR) and 95% confidence intervals (CI). Sampling weights and complex survey designs were applied to all analyses.

The sample was randomized into two equal groups to create a developmental and validation data set, pooling participants across all survey years (~ 22,150 each). The associations of individual characteristics with albuminuria were examined with odds ratios (OR) and 95% confidence intervals (CI), adjusting for covariates. Three models were developed. The first model included clinically available information on demographics and clinical characteristics: age, sex, race/ethnicity, current smoking status, prediabetes, high BP, and history of CVD. The second model added biochemical tests and outpatient medications: BMI ≥ 30 kg/m^2^, uric acid, low eGFR, low HDL, taking an ACEi, ARB, or a statin. The third model included significant interactions derived from investigations employing random forest machine learning techniques.^27^ Machine learning models determine the best fit for prediction and do not consider biological relevance, therefore some of the elucidated interactions may not have been considered with traditional model building.

To assess model fit, we examined C-statistics and developed receiver operator curves (ROC) for each of the three models and evaluated the sensitivity vs. 1-specificity for potential screening probability cut-points at increments of 0.02. For Model 3, we also evaluated the percent of the population that would need to be screened to detect a proportion of the albuminuria by cut point. A sensitivity analysis was employed among participants of the 2009–2010 sample who had two measurements of UACR. All three models were estimated, with corresponding C-statistics, as well as sensitivity and specificity for different cut-points.

All analyses were conducted using SAS 9.4 (SAS Institute Cary, North Carolina). NHANES data are publicly available for use by researchers and do not require IRB approval.

## RESULTS

### Descriptive Characteristics

**Figure 1:**
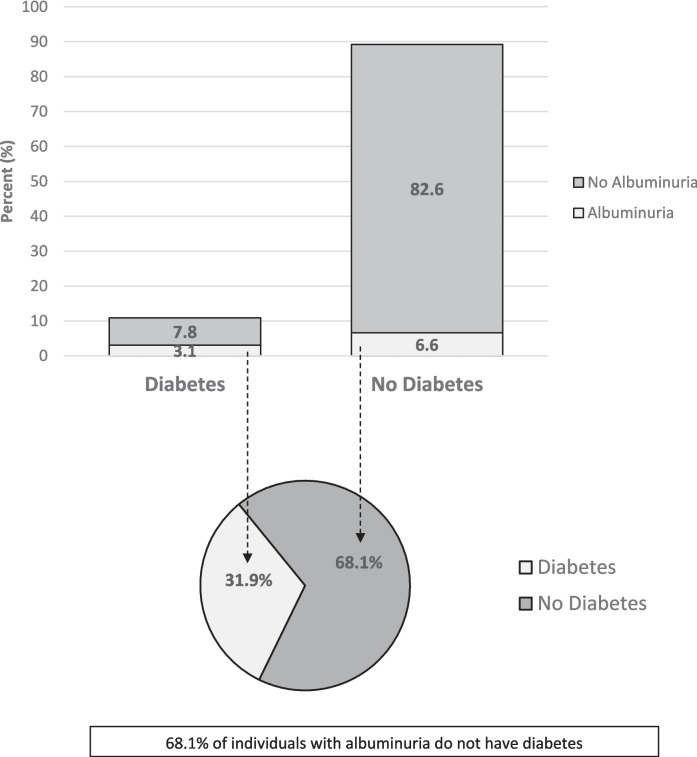
Overall prevalence and distribution of albuminuria by diabetes, among U.S. adults. Data source: national health and nutrition examination survey (NHANES), 1999-March 2020 participants aged 18 & older.

The overall prevalence of albuminuria among individuals with and without diabetes was 9.7% Although the prevalence of albuminuria is proportionately higher among those with diabetes, two-thirds of US adults with albuminuria do not have diabetes (Fig. 1). Figure 1 also shows the relative proportions of those with albuminuria among diabetic and nondiabetic individuals.

Among US adults, the mean age was 44.3 years (95% CI: 43.9–44.6), 48.4% were male, 69.0% were Non-Hispanic White, 61.1% had a college education, 81.9% had health insurance, and 32.3% had obesity with average BMI of 28.30 kg/m^2^ (Table 1). A vast majority of the population was either a current smoker (47.6%) or former smoker (38.8%), with a lower prevalence of low eGFR (4.8%), prediabetes (19.8%), diagnosis or medication for hypertension (30.6%), an elevated measured BP (25.7%), CVD (6.3%), and a history of at least one kidney stone (8.4%). A small proportion of the population was taking an ACE inhibitor (7.3%), an ARB (4.2%), or a statin (10.8%).

**Table 1:** Univariate Associations with Albuminuria among U.S. Adults without Diabetes (*N* = 44,322) Included in NHANES, 1999–2020*

Variable	Mean or % (95% CI for Mean)	OR	95% CI	*p*-value
Age (per year)	44.3 (43.9 – 44.6)	1.03	1.02—1.03	< 0.001
18 – 24 years	13.8% (13.1%—14.5%)	1.19	1.05—1.35	0.008
25 – 64 years	72.1% (71.4%—72.8%)	1.00	-	Ref
65 – 74 years	8.4% (7.9%—8.9%)	2.14	1.86—2.46	< 0.001
75 + years	5.7% (5.4%—6.1%)	4.81	4.31—5.38	< 0.001
Male (OR vs. female)	48.4% (47.9%—48.9%)	0.67	0.61—0.73	< 0.001
Race/Ethnicity:				
Non-Hispanic White	69.0% (67.1%—71.0%)	1.00	-	Ref
Non-Hispanic Black	10.2% (9.1%—11.2%)	1.36	1.23—1.51	< 0.001
Mexican American	8.2% (7.2%—9.2%)	1.10	0.96—1.25	0.163
Other Hispanic	5.7% (4.9%—6.5%)	1.02	0.81—1.24	0.987
Other non-Hispanic	12.6% (11.6%—13.6%)	1.13	0.96—1.33	0.151
College Education + (yes vs. no)	61.1% (59.8%—62.5%)	0.69	0.62—0.76	< 0.001
BMI (OR per 1 kg/m^2^)	28.2 (28.1–28.4)	1.01	1.00—1.02	0.019
BMI ≥ 30 kg/m^2^ (yes vs. no)	32.3% (31.5%—33.0%)	1.20	1.10—1.31	< 0.001
Current Smoker (yes vs. no)	47.6% (46.3%—49.0%)	1.19	0.95—1.49	0.122
Former Smoker (yes vs. no)	38.8% (37.4%—40.1%)	1.34	1.06—1.70	0.013
Health Insurance (yes vs. no)	81.9% (81.0%—82.8%)	1.11	0.99—1.24	0.075
Low eGFR^a^ (yes vs. no)	4.8% (4.5%—5.2%)	4.58	4.02—5.21	< 0.001
Prediabetes^b^ (yes vs. no)	19.8% (19.3%—20.4%)	1.81	1.65—1.99	< 0.001
Diagnosed Hypertension (yes vs. no)	30.6% (29.9%—31.4%)	2.51	2.29—2.75	< 0.001
High Blood Pressure^c^ (yes vs. no)	25.7% (25.0%—26.5%)	4.36	3.97—4.79	< 0.001
Cardiovascular Disease (yes vs. no)	6.3% (6.0%—6.6%)	2.82	2.50—3.19	< 0.001
Total cholesterol (OR per 50 mg/dl)	195.3 (194.5—196.0)	1.01	0.99—1.02	0.126
HDL (OR per 10 mg/dl)	53.9 (53.6—54.3)	1.04	1.01—1.07	0.015
Low HDL^d^ (yes vs. no)	6.7% (6.3%—7.1%)	1.40	1.19—1.66	< 0.001
High Uric Acid^e^ (yes vs. no)	13.3% (12.8%—13.8%)	2.05	1.83—2.28	< 0.001
Taking ACE Inhibitor (yes vs. no)	7.3% (6.9%—7.6%)	2.11	1.86—2.40	< 0.001
Taking ARB (yes vs. no)	4.2% (3.9%—4.5%)	1.97	1.68—2.33	< 0.001
Taking Statin (yes vs. no)	10.8% (10.2%—11.3%)	1.69	1.52—1.89	< 0.001
Kidney Stone^f^ (yes vs. no)	8.4% (7.9%—8.9%)	1.33	1.12—1.57	0.001
Household Income:				
Missing	21.9% (20.6%—23.3%)	1.44	1.23—1.69	< 0.001
< 45 K	35.7% (34.4%—37.0%)	1.84	1.60—2.12	< 0.001
45—75 K	17.3% (16.6%—18.1%)	1.25	1.05—1.48	0.011
75 K +	25.0% (23.7%—26.4%)	1.00	-	Ref
Moderate activity (yes vs. no)	64.3% (63.4%—65.2%)	0.71	0.64—0.80	< 0.001
Vigorous activity (yes vs. no)	43.0% (42.0%—44.0%)	0.59	0.53—0.65	< 0.001

Data Source: National Health and Nutrition Examination Survey (NHANES), 1999-March 2020 participants aged 18 & older, *N* = 44,322

^a^Low eGFR: CKD_EPI_eGFR < 60

^b^Prediabetes: HbA1c > = 5.7 and < 6.5

^c^High blood pressure > 140/90

^d^ HDL < 30 for males and < 40 for females

^e^For female uric acid > 6.1 and male uric acid > 7.2

^f^Kidney stone data only available from 2007 to March 2020

*Abbreviations: ACE inhibitor,* angiotensin-converting enzyme inhibitor;* ARB, angiotensin receptor blocker*

### Predictors of Albuminuria

By examining age through unadjusted logistic regression, the relationship showed a J-shape with the oldest age groups significantly associated with higher odds of albuminuria; for ages 65–74 years (*p* < 0.001), for ages 75 + years (*p* < 0.001), and 18–24 years (*p* = 0.008), compared with the 25–64 years age group (ref). Compared with their counterparts, men were less likely to have albuminuria (*p* < 0.001), and non-Hispanic Black adults were more likely to have albuminuria (*p* < 0.001). Having a college education was associated with lower odds of albuminuria (*p* < 0.001) and lower income < $45 K was associated with greater odds of albuminuria vs. $75 K + , *p* < 0.001.

Multiple co-morbidities were strongly associated with albuminuria. A low eGFR (OR = 4.58; *p* < 0.001) and an elevated blood pressure measurement (OR = 4.36; *p* < 0.001) were each associated with over four times the odds of albuminuria. Hypertension and cardiovascular disease were associated with over two times the odds of albuminuria. Prediabetes (OR = 1.81; *p* < 0.001) and nephrolithiasis (OR = 1.33; *p* = 0.001), low HDL (OR = 1.4; *p* < 0.001) and high uric acid (OR = 2.05; *p* < 0.001) were also associated with albuminuria. Each of the three medication classes were associated with albuminuria: being on an ACE inhibitor (*p* < 0.001), an ARB inhibitor (*p* < 0.001), or a statin (*p* < 0.001). Higher levels of self-reported physical activity, both moderate and vigorous were associated with lower odds of albuminuria (*p* < 0.001).

Results for the developmental data are included in Table 2 (see Supplemental Table 1 for the Validation dataset results). The c-statistic value for model 1 was 0.739 for the developmental model, and 0.734 for the validation model consistent with reasonable classification. Model 2 included all variables from Model 1 and added BMI ≥ 30 kg/m^2^, uric acid, low eGFR, low HDL, and three separate variables regarding medication use (taking an ACEi, taking an ARB, and taking a statin). The c-statistics were 0.749 for the developmental model and 0.742 for the validation model, like Model 1. Model 3 included all variables in Model 2 and added the interaction terms. The c-statistics were 0.756 for the developmental model and 0.752 for the validation model for Model 3. In comparison to Model 1 and Model 2, Model 3 showed a slightly higher c-statistic, by adding interaction terms.

**Table 2:** Multivariable Associations between Participant Characteristics and Albuminuria, Development Data Set

	Model 1 (*N* = 22,216) C-Statistics = 0.739			Model 2 (*N* = 22,216) C-Statistics = 0.749			Model 3 (*N* = 22,216) C-Statistics = 0.756		
Measure	Odds Ratio	95% Confidence Limits	*p*-value	Odds Ratio	95% Confidence Limits	*p*-value	Odds Ratio	95% Confidence Limits	*p*-value
Age 18—24 years	1.69	1.32—2.17	< 0.001	1.65	1.29—2.12	< 0.001	-	-	-
Age 25—64 years	1.00	-	ref	1.00	-	ref	-	-	-
Age 65—74 years	1.28	1.02—1.60	0.030	1.30	1.02—1.66	0.034	-	-	-
Age 75 + years	2.55	2.05—3.16	< 0.001	2.22	1.71—2.89	< 0.001	-	-	-
Male (vs. female)	0.68	0.60—0.77	< 0.001	0.69	0.61—0.79	< 0.001	-	-	-
Race/Ethnicity (vs. Non-Hispanic White)									
Non-Hispanic Black	1.29	1.10—1.51	0.002	1.26	1.07—1.48	0.005	-	-	-
Mexican American	1.72	1.42—2.09	< 0.001	1.71	1.41—2.07	< 0.001	1.69	1.39—2.05	< 0.001
Other Hispanic	0.88	0.63—1.23	0.457	0.87	0.62—1.23	0.432	0.87	0.62—1.21	0.396
Other Non-Hispanic	1.53	1.19—1.96	< 0.001	1.52	1.19—1.95	< 0.001	1.52	1.19—1.94	0.001
Current Smoker (yes vs. no)	1.40	1.15—1.70	< 0.001	1.38	1.13—1.68	0.002	1.36	1.12—1.66	0.002
Prediabetes^b^ (yes vs. no)	1.17	1.01—1.35	0.037	1.17	1.01—1.36	0.037	-	-	-
High BP^c^ (yes vs. no)	4.11	3.44—4.90	< 0.001	4.37	3.60—5.30	< 0.001	-	-	-
CVD (yes vs. no)	1.24	1.03—1.49	0.026	1.31	1.08—1.60	0.007	1.23	1.01—1.50	0.046
BMI ≥ 30 kg/m^2^ (yes vs. no)				1.01	0.87 – 1.16	0.991	-	-	-
Uric acid^d^ (per 1 mg/dl)				1.44	1.19—1.74	< 0.001	-	-	-
Low eGFR^a^ (yes vs. no)				1.71	1.35—2.17	< 0.001	1.76	1.39—2.23	< 0.001
Low HDL (yes vs. no)				1.25	0.98—1.60	0.070	1.26	0.99—1.60	0.070
Taking ACEi (yes vs. no)				0.70	0.58—0.84	< 0.001	0.70	0.59—0.84	< 0.001
Taking ARB (yes vs. no)				0.65	0.48—0.87	0.004	0.65	0.49—0.88	0.005
Taking Statin (yes vs. no)				0.73	0.60—0.90	0.003	0.72	0.59—0.88	0.002
Male High BP (vs. Female no High BP)							5.08	2.70—9.58	< 0.001
Female High BP (vs. Female no High BP)							3.17	2.42—4.15	< 0.001
No Prediabetes High Uric acid (vs. no Prediabetes and no High Uric acid)							1.83	1.43—2.34	< 0.001
Male Age 18—25 years (vs. Female Age 25—64 years)							0.47	0.29—0.77	0.003
Male Age 25—64 years (vs. Female Age 25—64 years)							0.29	0.21—0.41	< 0.001
Male Age 65—74 years (vs. Female Age 25—64 years)							0.50	0.35—0.71	0.0001
Female Age 18—25 years (vs. Female Age 25—64 years)							1.81	1.31—2.52	0.001
Female Age 75 + years (vs. Female Age 25—64 years)							1.86	1.36—2.54	0.0001
Black Age 25 **–** 64 years (vs. Non-Black Age 25–64 years)							1.92	1.55 – 2.39	0.005

The data was divided in half for developmental and validation sets. Models 1–3 below show increasing numbers of characteristics, which are related to increasing measures of prediction. The optimal measures for the best set of characteristics were identified to achieve the best selections for albuminuria prediction

Data Source: National Health and Nutrition Examination Survey (NHANES), 1999-March 2020 participants aged 18 & older

^a^Low eGFR: CKD_EPI_eGFR < 60;

^b^Prediabetes: HbA1c > = 5.7 and < 6.5;

^c^High blood pressure > 140/90;

^d^For female uric acid > 6.1 and male uric acid > 7.2;

Abbreviations: ACE inhibitor, angiotensin-converting enzyme inhibitor; ARB, angiotensin receptor blocker

In the sensitivity analysis, using the full-model variable list (Model 3) among individuals with two measurements of albuminuria, we found consistent results, shown in Supplemental Table 3. The c-statistic was higher for this model, reflecting an even higher predictive ability to identify individuals with two confirmed measurements of high UACR. Due to the variability in UACR, though, the original may be preferred for use in screening, to attempt to capture any abnormally high measurements.

**Figure 2:**
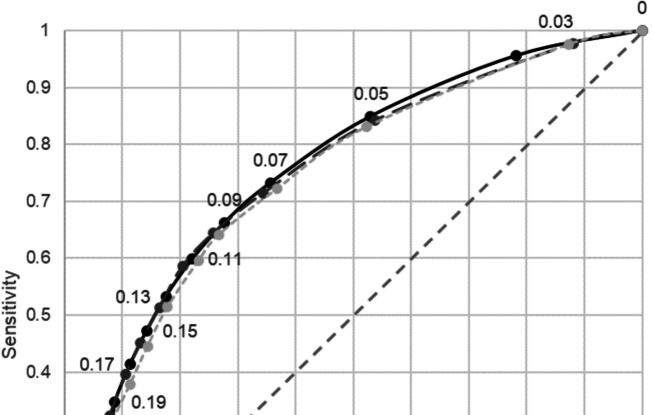
ROC curve for predicting albuminuria in development data Set. Figure 2 displays ROC curves for predicting albuminuria for model 1, 2, and 3. Cut-points are denoted along each curve as indicated with markers and corresponding cut-point value.

**Figure 3:**
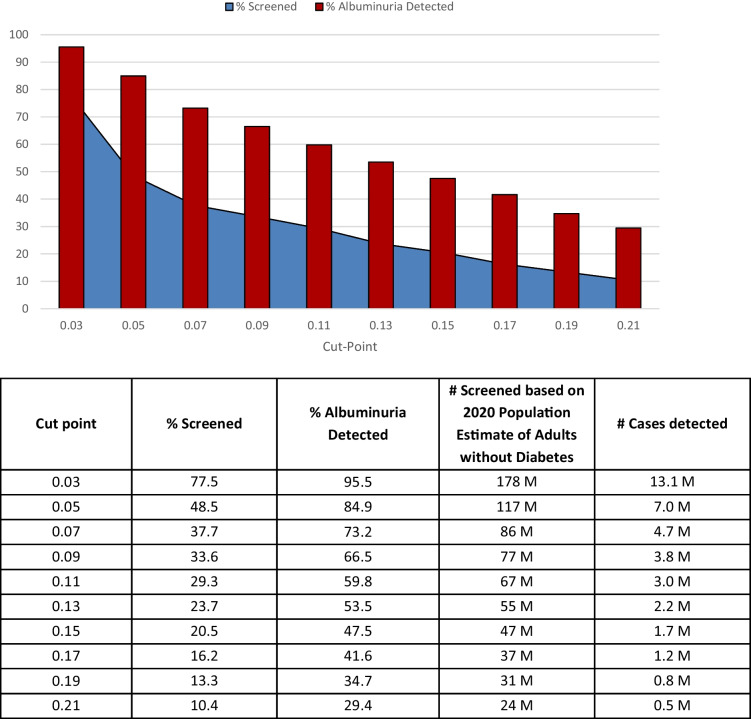
Percent of population screened by model 3 and proportion of albuminuria detected by cut point. * Estimates are conservative, as they applied the overall prevalence of albuminuria among individuals without diabetes during the study period and these rates have been increasing over time.

Figure 2 compares the ROC curves for predicting albuminuria for these three models and supplemental Table 2 shows the sensitivity and 1-specificity per cut-point for models 1, 2, and 3. The sensitivity and 1-specificity for all models at all cut-points is comparable despite the inclusion of more variables and interactions. Figure 3 displays the percentage of the population screened and proportion of albuminuria detected by cut point for Model 3. The blue graph displays the percentage of the population screened to detect the percentage of albuminuria detected by the red bars. For example, employing a cut-point of 0.05 for screening would require just under half of the non-diabetic population to be screened, but would detect 85% of individuals with albuminuria, i.e., approximately 1 in 16 of the high-risk individuals screened. Moving the cut point up to 0.07 would require just over a third of the population to be screened and detect approximately 3/4 of individuals with albuminuria and correctly detect albuminuria in 1 out 18 high-risk individuals screened.

### Sensitivity Analysis using Two Measurements of Albuminuria

Consistent results were found when repeating the analyses among a sample of participants with two measurements of albuminuria available, although due to the much smaller (1/10) sample size, levels of significance were reduced. A J-shaped association between age and odds of albuminuria persisted, as well as a higher likelihood for Mexican Americans, smoking, prediabetes, high blood pressure, CVD, obesity, low eGFR, and high uric acid. Interesting differences, although not statistically significant, were higher odds of albuminuria for males vs. females and no difference in odds of albuminuria for Black vs. White participants. Results of these models are displayed in Supplemental Table 2.

C-statistics for these models were consistently higher (0.795, 0.812, and 0.833, respectively), likely due to reduced error in the dependent variable with use of two measures indicating albuminuria. Employing the same cut-point of a 5% predicted probability of albuminuria for screening yielded a sensitivity of 0.59 and specificity of 0.79.

### Risk Assessment Calculator

Appendix 1 displays these three predictive models as risk assessment calculators. Sheet 1 displays Model 1, Sheet 2 displays Model 2, and Sheet 3 displays Model 3. By inputting the data for a given individual in a chosen model, assuming “1” indicates “yes” and “0” indicates “no” for any most listed parameters and “1” indicates “male” and “0” indicates “female” for sex, our calculators output the probability of albuminuria for that individual using each of these previously described models. A calculator for Model 3 is given for both cohorts (the full cohort from 1999-2020) and for the cohort with two UACR measurements (2009-2010).

## DISCUSSION

In this large cross-sectional study, older age, male gender, Non-Hispanic Black race, Mexican American ethnicity, cigarette smoking, prediabetes, high blood pressure, and cardiovascular disease were strongly associated with albuminuria. We demonstrated that a reasonable risk score-based prediction framework could be developed to determine whom to screen for albuminuria in the non-diabetic population. Integrating individual-level patient characteristics strongly associated with higher odds of albuminuria, we developed three multivariable models to test the predicted probability for albuminuria. We created one model with more readily available predictors and two other models with a broader set of predictors. The three models yielded reasonably good c-statistics, and the addition of more variables did not considerably affect concordance. Based on Model 3, if we screened everyone with a 7% or higher predicted probability of having albuminuria, we could detect 73.2% of individuals with albuminuria by screening 37.7% of the non-diabetic population.

Many strategies have been proposed to screen for albuminuria. De Jong and Curhan explored initiating a screening program for albuminuria in high-risk individuals.^4^ Boulware et al. 2003 explored the value of periodic dipstick screening and found that this method was not cost-effective unless directed specifically toward high-risk groups or conducted at infrequent intervals of 10 years.^17^ Hoerger et al. found that microalbuminuria screening is only cost effective for patients with diabetes or hypertension as well or if conducted at longer intervals at established physician visits.^18^ Targeting screening to high-risk populations has shown potential in cost effectiveness analyses to identify more feasibly those with impaired kidney function.

Early identification of albuminuria has significant diagnostic and therapeutic implications. The presence of albuminuria may be and earlier sign of kidney damage, which can arise due to hyperglycemia, hypertension, obesity, smoking, etc., prompting physician and patients to place greater emphasis on optimization of health behaviors and medical therapies. Albuminuria could also be the initial manifestation of nephrotic or nephritic syndrome requiring more specialized investigation and treatment. Treatment with angiotensin-converting enzyme inhibitors (ACEIs) and angiotensin II receptor blockers (ARBs) has been associated with reduced urine protein excretion and a slower decline of GFR among both diabetic and non-diabetic individuals.^19–23^ In populations with CKD and albuminuria, large clinical trials have consistently demonstrated that sodium/glucose cotransporter-2 inhibitors (SGLT2i) and non-steroidal mineralocorticoid receptor antagonists such as finerenone, can significantly slow progression of CKD and reduce mortality from renal and cardiovascular causes.^24,25^ Earlier detection can lead to earlier medical optimization of underlying kidney disease which can in turn slow progression.^1–3^

Screening for albuminuria in the non-diabetic population has previously been explored. Boulware et al. 2003 and others suggest that screening is not cost-effective unless specifically targeted toward high-risk populations.^17,28–32^ This notion has been attributed to the low prevalence and incidence of proteinuria, and therefore few cases of preventable ESKD, resulting in a minimal gain in quality adjusted life years to balance the costs.^17^ Some studies have suggested targeted screening in high-risk populations based on race/ethnicity or comorbidities.^17,28–32^ Machine learning algorithms have been explored to predict the development of albuminuria among those with diabetes.^33,34^ To our knowledge, ours is the first study to develop predictive models for albuminuria aimed at targeting high risk populations among those who are non-diabetic.

Consistent with prior work, we found that hypertension and high blood pressure are strongly associated with albuminuria,^35,36^ though current guidelines differ in their recommendations regarding screening for albuminuria in this population. The 2013 American College of Physicians recommends against routine testing and monitoring of albuminuria especially in those already taking an ACEi or an ARB.^16^ However, the 2018 European Society of Cardiology recommends an annual albumin creatinine ratio in those with hypertension^15^ and the 2017 American College of Cardiology and 2020 International Society of Hypertension suggest that serial testing for ACR can add value as a part of optimal care.^37^ The discrepancy in these guidelines may be because ACEi/ARBs are typically recommended as initial therapy for hypertension, and therefore the prevailing notion (which represents a knowledge gap regarding the value of albuminuria for risk stratification) is that identification of albuminuria might not change clinical management. Further, there is emerging evidence that newer therapies e.g., sodium/glucose cotransporter-2 inhibitors (SGLT2i) and non-steroidal mineralocorticoid receptor antagonists such as finerenone are indicated in patients with CKD regardless of the presence or absence of diabetes and can provide additional benefit to those already receiving ACEi/ARBs, along with close attention to lifestyle changes. Recommendations for more widespread screening among those with key risk factors for kidney disease are emerging.^3,24,25^

We also observed multiple individual-level associations with albuminuria that were consistent with prior literature. Older age was significantly associated with higher risks of albuminuria, increased mortality, and cardiovascular risk in these populations compared to those without albuminuria, especially in those older than 65 years.^38–41^ Additionally, male sex was associated with lower odds of albuminuria compared to female sex. Previous work using NHANES data has also demonstrated a higher prevalence of albuminuria in females (vs males), but there is some concern that the use of ACR as a measurement for albuminuria may be biased toward being higher in females, given that creatinine excretion is lower than in males.^6,38,42,43^ In multivariable analyses, we noted higher odds of albuminuria among non-Hispanic Blacks, Mexican Americans, and Other non-Hispanic populations including Asians, Native Americans, and Pacific Islanders. In the general population as well as the subset who have diabetes, Blacks and Hispanics have been shown to have higher odds of microalbuminuria in comparison to White populations.^38,44–46^ Smoking has been associated with albuminuria and abnormal kidney function in both those without diabetes and those with diabetes.^47–52^ The mechanism by which smoking leads to kidney damage is not clear but is likely multifactorial, including progressive renal atherosclerosis and oxidative stress with higher levels of toxic compounds including cadmium, copeptin, and cotinine. Microalbuminuria is strongly associated with diabetes and a significant risk factor for cardiovascular mortality.^7,19,23,53–58^ In our study, earlier stages of glucose intolerance or prediabetes were also associated with albuminuria, consistent with prior work.^59–64^

Our approach utilized machine learning algorithms which excel in analyzing large volumes of complex medical data and extracting patterns to inform our models. Machine learning also enabled us to develop models that can provide more personalized recommendations to clinicians. There are a few limitations. Machine learning can extrapolate biases in the data and can have limited generalizability and performance with new data. Additionally, this was a cross-sectional study and therefore does not allow examination of the temporal relationship between risk factors and albuminuria. Future work in longitudinal studies could evaluate the incidence of albuminuria to better understand this temporal relationship. Also, we defined albuminuria using urine albumin to creatinine ratio, though this measurement has the potential to be influenced by lower creatinine excretion in women and in those with age-related muscle loss.^43^ Finally, this study is limited by the fact that in NHANES only a single measure of albumin to creatinine ratio is available in those undergoing urine testing, whereas in clinical practice, repeat urine testing is advisable to confirm the persistence of albuminuria. To address this limitation, we also present data on a subsample (2009–2010), which had two measures of albuminuria.

Despite these limitations, our study has several notable strengths. We utilized a large nationally representative sample of the US population. We were able to identify multiple clinically relevant factors associated with albuminuria that are consistent with published literature. We developed three reasonably strong models to predict albuminuria, one incorporating more readily available variables and two with a broader array of variables and transformed our models into risk assessment calculators that are included in Appendix 1 for use in future work.

Future analyses and prospective studies are needed to replicate and validate our findings in clinical practice. While we considered screening cut-points of 5% and 7% for routine care, we need to further determine appropriate cut-points for screening based on detection probabilities and their implications. There are also multiple alternatives to integrating this tool into medical care, and the benefits and risks of each of these options need to be further explored in pragmatic, prospectively designed studies. Future investigations would need to assess the utility of this tool to increase detection of albuminuria as an early marker of CKD (and cardiovascular disease), and the longitudinal impact on diagnosis, treatment, and patient outcomes.

## Supplementary Information

Below is the link to the electronic supplementary material.

Supplementary file1 (ZIP 53.8 KB)

## Data Availability

Data are used for this analysis are publicly available from the National Health and Nutrition Examination Survey website: https://www.cdc.gov/nchs/nhanes/index.htm.
