# Supplementary material for: Using Risk Assessment to Improve Screening for Albuminuria among US Adults without Diabetes
Source: J Gen Intern Med. 2024 Nov 18;40(13):3159–69. doi: 10.1007/s11606-024-09185-9 (PMC12508371; doi:10.1007/s11606-024-09185-9)
Supplement: Supplementary file 1 — Supplementary file1 (ZIP 53.8 KB) [file 11606_2024_9185_MOESM1_ESM.zip › 11606_2024_9185_MOESM1_ESM.docx]

**Table 1: Patient Characteristics by Randomized Development and Validation Data**

| **Measure** | **Percent** | |
| --- | --- | --- |
|  | **Developmental (n=22,106)** | **Validation (n=22,106)** |
| Age: (%) |  |  |
| 18 - 25 years | 12.5 | 12.2 |
| 25 - 64 years | 70.5 | 70.7 |
| 65 - 74 years | 10.1 | 10.1 |
| 75 + years | 6.9 | 7.0 |
| Male (%) | 48.3 | 48.5 |
| Race/Ethnicity: (%) |  |  |
| Non-Hispanic White | 67.7 | 68.2 |
| Non-Hispanic Black | 10.9 | 10.5 |
| Mexican American | 8.4 | 8.3 |
| Other Hispanic | 6.0 | 5.8 |
| Other non-Hispanic | 13.0 | 13.0 |
| Current Smoker (%) | 20.4 | 20.6 |
| Prediabetes ^b^ (%) | 20.1 | 19.9 |
| High BP ^c^ (%) | 32.9 | 33.7 |
| CVD (%) | 8.4 | 8.6 |
| BMI ≥ 30 kg/m^2^ (%) | 35.1 | 35.5 |
| High acid^d^ (%) | 14.4 | 14.5 |
| Low eGFR^a^ (%) | 6.5 | 6.2 |
| Low HDL (%) | 7.3 | 7.6 |
| Taking ACEi (%) | 10.2 | 10.5 |
| Taking ARB (%) | 5.5 | 5.5 |
| Taking Statin (%) | 13.9 | 15.1 |

**Supplemental Table 2. Multivariable Associations between Participant Characteristics and Albuminuria, Validation**

| **Measure** | **Model 1 (N = 22,106)**  **C-Statistics = 0.734** | | | **Model 2 (N = 22,106)**  **C-Statistics = 0.741** | | | **Model 3 (N = 22,106)**  **C-Statistics = 0.752** | | |
| --- | --- | --- | --- | --- | --- | --- | --- | --- | --- |
|  | **Odds Ratio** | **95% Confidence Limits** | **p-value** | **Odds Ratio** | **95% Confidence Limits** | **p-value** | **Odds Ratio** | **95% Confidence Limits** | **p-value** |
| Age 18 - 25 years | 1.71 | 1.36 - 2.14 | <0.001 | 1.67 | 1.34 - 2.09 | <0.001 | - | - | - |
| Age 25 - 64 years | 1.00 | - | ref | 1.00 | - | ref | - | - | - |
| Age 65 - 74 years | 1.26 | 1.03 - 1.55 | 0.027 | 1.28 | 1.03 - 1.58 | 0.025 | - | - | - |
| Age 75 + years | 2.44 | 2.02 - 2.96 | <0.001 | 2.17 | 1.77 - 2.67 | <0.001 | - | - | - |
| Male (vs. female) | 0.64 | 0.56 - 0.73 | <0.001 | 0.65 | 0.56 - 0.74 | <0.001 | - | - | - |
| Race/Ethnicity (vs. Non-Hispanic White) |  | | |  |  |  |  |  |  |
| Non-Hispanic Black | 1.41 | 1.20 - 1.64 | <0.001 | 1.38 | 1.19 - 1.62 | <0.001 | - | - | - |
| Mexican American | 1.46 | 1.21 - 1.77 | <0.001 | 1.46 | 1.21 - 1.76 | <0.001 | 1.46 | 1.21 - 1.77 | 0.0002 |
| Other Hispanic | 1.3 | 0.99 - 1.70 | 0.062 | 1.28 | 0.98 - 1.67 | 0.071 | 1.32 | 1.01 - 1.74 | 0.047 |
| Other Non-Hispanic | 1.2 | 0.96 - 1.51 | 0.108 | 1.21 | 0.97 - 1.51 | 0.098 | 1.17 | 0.93 - 1.48 | 0.171 |
| Current Smoker (yes vs. no) | 1.39 | 1.16 - 1.67 | <0.001 | 1.37 | 1.14 - 1.65 | <0.001 | 1.35 | 1.11 - 1.65 | 0.003 |
| Prediabetes^b^ (yes vs. no) | 1.02 | 0.88 - 1.18 | 0.840 | 1.03 | 0.88 - 1.19 | 0.733 | - | - | - |
| High BP^c^ (yes vs. no) | 4.1 | 3.36 - 5.01 | <0.001 | 4.36 | 3.56 - 5.35 | <0.001 | - | - | - |
| CVD (yes vs. no) | 1.58 | 1.33 - 1.87 | <0.001 | 1.61 | 1.35 - 1.91 | <0.001 | 1.56 | 1.32 - 1.85 | <0.001 |
| BMI ≥ 30 kg/m^2^ (yes vs. no) |  |  |  | 0.96 | 0.83 – 1.11 | 0.556 | - | - | - |
| Uric acid^d^ (per 1 mg/dl) |  |  |  | 1.30 | 1.11 - 1.52 | 0.002 | - | - | - |
| Low eGFR^a^ (yes vs. no) |  |  |  | 1.56 | 1.23 - 1.97 | <0.001 | 1.56 | 1.25 - 1.96 | 0.0001 |
| Low HDL (yes vs. no) |  |  |  | 1.22 | 0.96 - 1.55 | 0.098 | 1.21 | 0.95 - 1.54 | 0.122 |
| Taking ACEi (yes vs. no) |  |  |  | 0.75 | 0.60 - 0.94 | 0.012 | 0.74 | 0.59 - 0.92 | 0.008 |
| Taking ARB (yes vs. no) |  |  |  | 0.68 | 0.53 - 0.88 | 0.004 | 0.68 | 0.53 - 0.88 | 0.004 |
| Taking Statin (yes vs. no) |  |  |  | 0.84 | 0.71 - 1.00 | 0.056 | 0.82 | 0.69 – 0.98 | 0.027 |
| Male High BP^c^ (vs. Female no High BP) |  |  |  |  |  |  | 6.01 | 3.48 - 10.41 | <0.001 |
| Female High BP^c^ (vs. Female no High BP) |  |  |  |  |  |  | 3.93 | 3.03 - 5.09 | <0.001 |
| No Prediabetes High Uric acid (vs. no Prediabetes and no High Uric acid) |  |  |  |  |  |  | 1.49 | 1.20 - 1.85 | 0.0004 |
| Male Age 18 - 25 years (vs. Female Age 25 - 64 years) |  |  |  |  |  |  | 0.40 | 0.25 - 0.62 | <0.001 |
| Male Age 25 **-** 64 years (vs. Female Age 25 - 64 years) |  |  |  |  |  |  | 0.32 | 0.24 - 0.43 | <0.001 |
| Female Age 18 - 25 years (vs. Female Age 25 - 64 years) |  |  |  |  |  |  | 2.13 | 1.61 - 2.81 | <0.001 |
| Female Age 75 + years (vs. Female Age 25 - 64 years) |  |  |  |  |  |  | 2.02 | 1.56 - 2.62 | <0.001 |
| Black Age 25 **–** 64 years (vs. Non-Black Age 25-64 years) |  |  |  |  |  |  | 1.83 | 1.20 - 2.79 | 0.005 |

*The data was divided in half for developmental and validation sets. This section evaluates whether the test and validation sets are similar.*

*Data Source: National Health and Nutrition Examination Survey (NHANES), 1999-March 2020 participants aged 18 & older.*

*^a^Low eGFR: CKD_EPI_eGFR < 60;*

*^b^Prediabetes: HbA1c >=5.7 and <6.5;*

*^c^High blood pressure > 140/90;*

*^d^For female uric acid >6.1 and male uric acid >7.2;*

*Abbreviations: ACE inhibitor,* angiotensin-converting enzyme inhibitor; *ARB, angiotensin receptor blocker.*

**Supplemental Table 3. Multivariable Associations between Participant Characteristics and Albuminuria, Two-Measures (2009-2010)**

|  | **Model 1 (N = 4,863)**  **C-Statistics = 0.795** | | | **Model 2 (N = 4,863)**  **C-Statistics = 0.812** | | | **Model 3 (N = 4,863)**  **C-Statistics = 0.833** | | |
| --- | --- | --- | --- | --- | --- | --- | --- | --- | --- |
| **Measure** | **Odds Ratio** | **95% Confidence Limits** | **p-value** | **Odds Ratio** | **95% Confidence Limits** | **p-value** | **Odds Ratio** | **95% Confidence Limits** | **p-value** |
| Age 18 - 25 years | 1.61 | 0.52 – 5.00 | 0.41 | 1.62 | 0.51 - 5.16 | 0.41 | - | - | - |
| Age 25 - 64 years | 1.00 | - | ref | 1.00 | - | ref | - | - | - |
| Age 65 - 74 years | 1.89 | 1.28 - 2.79 | 0.001 | 1.65 | 1.14 - 2.39 | 0.008 | - | - | - |
| Age 75 + years | 2.40 | 1.20 - 4.78 | 0.01 | 1.50 | 0.80 - 2.81 | 0.21 | - | - | - |
| Male (vs. female) | 1.26 | 0.85 - 1.86 | 0.26 | 1.21 | 0.82 - 1.80 | 0.34 | - | - | - |
| Race/Ethnicity (vs. Non-Hispanic White) |  | | |  |  |  |  |  |  |
| Non-Hispanic Black | 0.84 | 0.39 - 1.82 | 0.65 | 0.70 | 0.32 - 1.56 | 0.39 | - | - | - |
| Mexican American | 1.59 | 0.86 – 2.93 | 0.14 | 1.68 | 0.89 – 2.81 | 0.11 | 1.71 | 0.90 – 3.24 | 0.0002 |
| Other Hispanic | 0.92 | 0.35 - 2.44 | 0.87 | 0.97 | 0.39 – 2.44 | 0.95 | 0.91 | 0.35 – 2.34 | 0.047 |
| Other non-Hispanic | 1.76 | 0.58 - 5.34 | 0.32 | 1.67 | 0.59 – 4.74 | 0.34 | 1.68 | 0.56 – 4.99 | 0.171 |
| Current Smoker (yes vs. no) | 1.44 | 0.99 - 2.11 | 0.06 | 1.49 | 1.03 – 2.16 | 0.03 | 1.50 | 0.98 – 2.30 | 0.003 |
| Prediabetes^b^ (yes vs. no) | 1.22 | 0.66 - 2.25 | 0.52 | 1.28 | 0.66 – 2.49 | 0.46 | - | - | - |
| High BP^c^ (yes vs. no) | 10.4 | 5.07 - 21.2 | <0.001 | 12.7 | 5.81 – 27.6 | <0.001 | - | - | - |
| CVD (yes vs. no) | 1.02 | 0.64 - 1.62 | 0.95 | 1.11 | 0.65 - 1.88 | 0.70 | 0.98 | 0.55 - 1.76 | <0.001 |
| BMI ≥ 30 kg/m^2^ (yes vs. no) |  |  |  | 1.13 | 0.83 - 1.11 | 0.59 | - | - | - |
| High Uric acid^d^ (yes vs. no) |  |  |  | 1.32 | 0.69 - 2.54 | 0.40 | - | - | - |
| Low eGFR^a^ (yes vs. no) |  |  |  | 2.72 | 1.22 - 6.05 | 0.01 | 3.05 | 1.39 - 6.68 | 0.0001 |
| Low HDL (yes vs. no) |  |  |  | 0.47 | 0.21 - 1.05 | 0.07 | 0.46 | 0.20 - 1.05 | 0.122 |
| Taking ACEi (yes vs. no) |  |  |  | 0.59 | 0.25 - 1.40 | 0.23 | 0.61 | 0.25 - 1.46 | 0.008 |
| Taking ARB (yes vs. no) |  |  |  | 0.37 | 0.14 – 1.00 | 0.049 | 0.36 | 0.12 - 1.10 | 0.004 |
| Taking Statin (yes vs. no) |  |  |  | 0.91 | 0.48 - 1.70 | 0.76 | 0.90 | 0.47 - 1.72 | 0.027 |
| Male High BP^c^ (vs. Female no High BP) |  |  |  |  |  |  | 5.63 | 2.32 – 13.7 | <0.001 |
| Female High BP^c^ (vs. Female no High BP) |  |  |  |  |  |  | 7.88 | 3.59 – 17.3 | <0.001 |
| Prediabetes High Uric acid (vs. no Prediabetes and no High Uric acid) |  |  |  |  |  |  | 0.84 | 0.20 – 3.52 | 0.81 |
| Male Age 18 - 25 years (vs. Female Age 25 - 64 years) |  |  |  |  |  |  | 0.21 | 0.02 – 1.76 | 0.15 |
| Male Age 25 **-** 64 years (vs. Female Age 25 - 64 years) |  |  |  |  |  |  | 0.33 | 0.11 – 1.00 | 0.05 |
| Female Age 18 - 25 years (vs. Female Age 25 - 64 years) |  |  |  |  |  |  | 2.56 | 0.69 – 9.46 | 0.16 |
| Female Age 75 + years (vs. Female Age 25 - 64 years) |  |  |  |  |  |  | 0.78 | 0.33 – 1.84 | 0.58 |
| Black Age 25 **–** 64 years (vs. Non-Black Age 25-64 years) |  |  |  |  |  |  | 1.02 | 0.32 – 3.26 | 0.97 |

*The data was divided in half for developmental and validation sets. This section evaluates whether the test and validation sets are similar.*

*Data Source: National Health and Nutrition Examination Survey (NHANES), 1999-March 2020 participants aged 18 & older.*

*^a^Low eGFR: CKD_EPI_eGFR < 60;*

*^b^Prediabetes: HbA1c >=5.7 and <6.5;*

*^c^High blood pressure > 140/90;*

*^d^For female uric acid >6.1 and male uric acid >7.2;*

*Abbreviations: ACE inhibitor,* angiotensin-converting enzyme inhibitor; *ARB, angiotensin receptor blocker.*

**Supplemental Table 4. Sensitivity and 1-Specificity per Cut-Point for Model 1, 2, and 3**

| **Cut Point** | **Model 1** | | **Model 2** | | **Model 3** | | | |
| --- | --- | --- | --- | --- | --- | --- | --- | --- |
|  | **Sensitivity** | **Specificity** | **Sensitivity** | **Specificity** | **Sensitivity** | **Specificity** | **False +** | **False -** |
| 0.03 | 0.98 | 0.13 | 0.98 | 0.12 | 0.96 | 0.22 | 0.71 | 0.004 |
| 0.05 | 0.83 | 0.48 | 0.84 | 0.54 | 0.85 | 0.47 | 0.48 | 0.01 |
| *0.07* | *0.72* | *0.63* | *0.71* | *0.66* | *0.73* | *0.64* | *0.32* | *0.02* |
| 0.09 | 0.64 | 0.73 | 0.64 | 0.74 | 0.66 | 0.72 | 0.25 | 0.03 |
| 0.11 | 0.60 | 0.77 | 0.59 | 0.80 | 0.60 | 0.78 | 0.20 | 0.04 |
| 0.13 | 0.51 | 0.82 | 0.51 | 0.84 | 0.53 | 0.82 | 0.16 | 0.04 |
| 0.15 | 0.45 | 0.86 | 0.45 | 0.87 | 0.47 | 0.86 | 0.13 | 0.05 |
| 0.17 | 0.38 | 0.89 | 0.40 | 0.89 | 0.41 | 0.89 | 0.10 | 0.05 |
| 0.19 | 0.32 | 0.92 | 0.32 | 0.92 | 0.35 | 0.91 | 0.08 | 0.06 |
| 0.21 | 0.25 | 0.94 | 0.27 | 0.94 | 0.29 | 0.92 | 0.06 | 0.06 |
